# A Rare Case of Pulmonary Atresia with Ventricular Septal Defect with a Right Sided Aortic Arch and a Calcified Pulmonary AVM Presenting in an Adult without Cyanosis

**DOI:** 10.1155/2014/614647

**Published:** 2014-12-15

**Authors:** Devendra V. Kulkarni, Rahul G. Hegde, Ankit Balani, Anagha R. Joshi

**Affiliations:** Department of Radiology, Lokmanya Tilak Municipal Medical College and Lokmanya Tilak Municipal General Hospital, Sion, Mumbai 400022, India

## Abstract

Pulmonary atresia with ventricular septal defect (PA-VSD) with pulmonary arterial supply arising from the aorta representing large MAPCAs associated with a right sided aortic arch is an uncommon anomaly. Most of the patients succumb to severe respiratory compromise or congestive cardiac failure very early. We report the clinical details and imaging findings of a case of PA-VSD with a right sided aortic arch and a calcified pulmonary arteriovenous malformation (AVM) in a 21-year-old postpartum female with no previous episodes of cyanosis who was diagnosed as having a cardiac anomaly on echocardiography when she presented with breathlessness during the 8th month of the pregnancy.

## 1. Introduction

PA-VSD is extremely complicated class of congenital cardiac anomalies which presents with complete absence of any communication between the right ventricle and the pulmonary arteries with an associated ventricular septal defect and supply to the lung parenchyma via MAPCAs (major aortopulmonary collateral arteries) [1]. Its presentation in adult life without cyanosis is extremely rare. Our case also had a calcified pulmonary AVM which has not been reported in the literature.

It shares similarities with Tetralogy of Fallot (TOF) and has been considered a severe end of the spectrum of TOF. However, TOF involves pulmonary or infundibular stenosis and not pulmonary atresia. It also has a few similarities with truncus arteriosus and has been called pseudotruncus and truncus arteriosus type 4. However, truncus arteriosus involves a single channel arising from both the ventricular outflow tracts with both aorta and pulmonary artery arising from this channel. PA-VSD has now been recognized as a distinct entity with its typical findings and management issues [2].

## 2. Case Report

A 21-year-old female presented with a history of severe breathlessness in past during her 8th month of pregnancy. There was no previous history of similar complaints during the rest of her pregnancy, her childhood, and adolescence. General examination revealed grade I clubbing; however, there was no evidence of cyanosis. The resting oxygen saturation was mildly reduced at 92%. Systemic examination revealed systolic murmur in the precordium. Blood examinations reported increased ESR and leukocytes.

**Figure 1:**
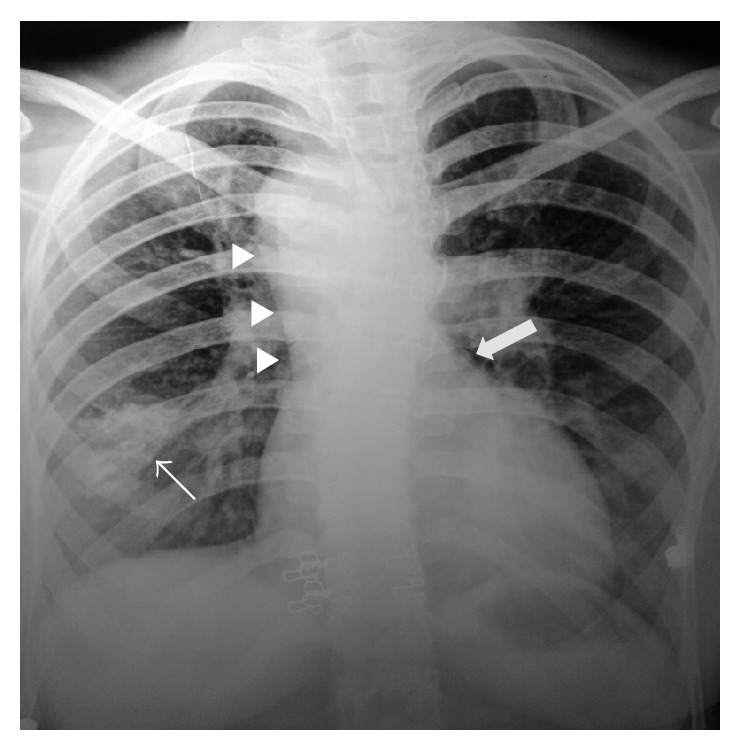
Chest radiograph: frontal view reveals left hilar prominence, right sided aortic arch (arrowheads), absent pulmonary conus (thick arrow), and a small ill-defined calcified nodular radio-opacity in the right lower zone of the lung (thin arrow).

Chest X-ray revealed hilar prominence on the left side, right sided aortic arch, absent pulmonary conus, and a small ill-defined calcified nodular radio-opacity in the right lower zone of the lung (Figure 1).

**Figure 2:**
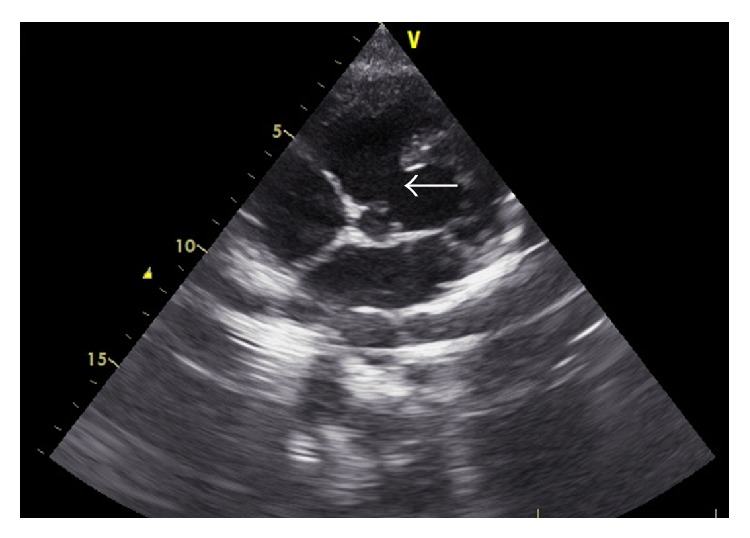
Transthoracic echocardiography: 5-chamber view shows a large malaligned nonrestrictive subaortic VSD (thin arrow).

**Figure 3:**
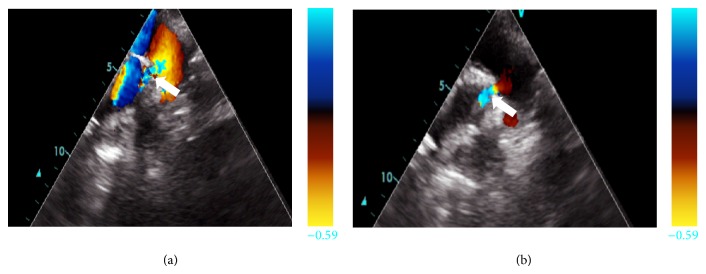
Colour doppler images from the transthoracic echocardiogram show right-to-left shunt through the subaortic VSD (arrow).

Transthoracic 2D echo revealed a single large malaligned nonrestrictive ventricular septal defect (VSD) with right-to-left (R-L) shunt, >50% overriding of aorta and pulmonary atresia (Figures 2 and 3). Suprasternal echocardiography revealed a MAPCA arising from the aorta.

For better delineation of the pulmonary arterial supply, a computed tomography pulmonary angiography (CTPA) was planned in the postpartum period to avoid fetal radiation.

**Figure 4:**
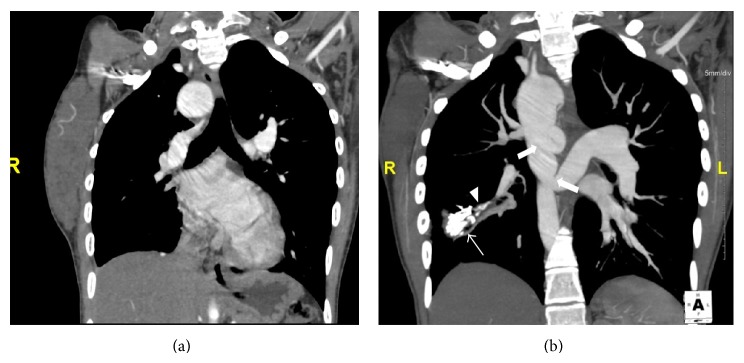
(a) and (b) Coronal CT pulmonary angiography: MIP (maximum intensity projection) images reveal a right sided aortic arch with large arterial channels arising from the left lateral aspect of the descending thoracic aorta (thick arrows) representing MAPCAs supplying the pulmonary parenchyma. Also seen is a calcified lesion (thin arrow) in the lower lobe of the right lung with a vascular pedicle (arrow head).

**Figure 5:**
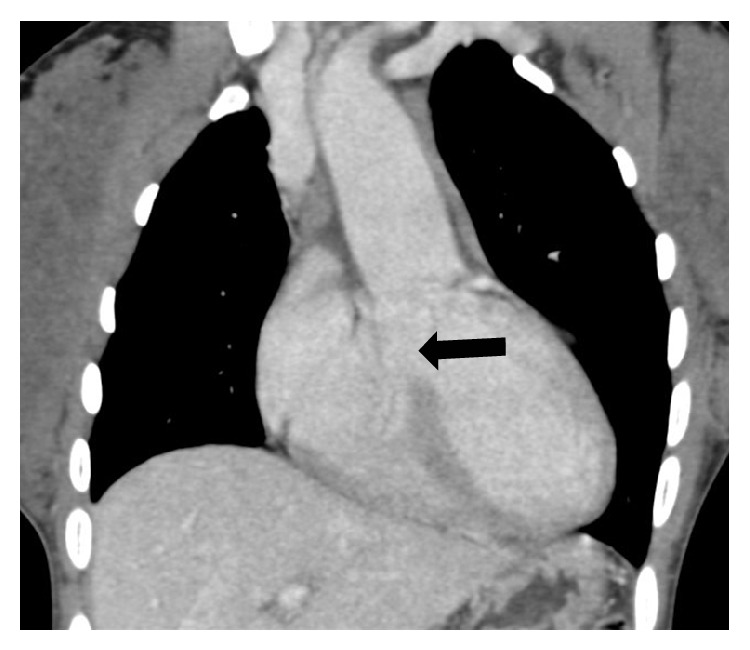
Coronal CT pulmonary angiography reveals a large subaortic VSD with overriding of the aorta (thick arrow).

**Figure 6:**
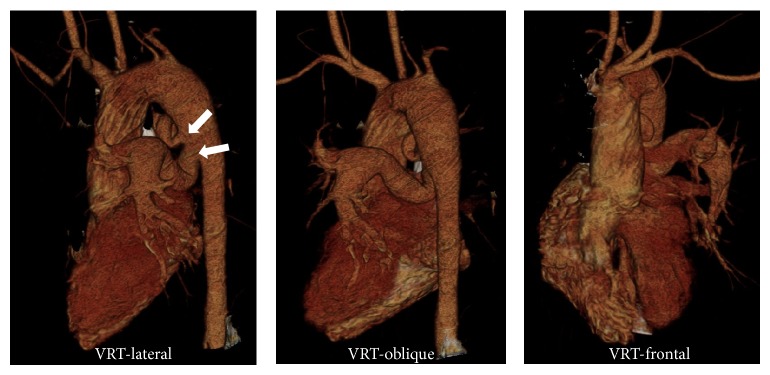
3D volume rendered images in lateral, oblique, and frontal projections showing large arterial channels arising from the left lateral aspect of the descending thoracic aorta (arrows) on the lateral projection representing MAPCAs. Overriding of aorta appreciated on the frontal projection.

CTPA revealed absent main pulmonary artery, right pulmonary artery, and left pulmonary artery with nonvisualisation of the right ventricular outflow tract indicating pulmonary atresia. Large arterial channels representing MAPCAs (2 on right and 1 on left) were seen arising from the aorta (one arterial channel arising from the descending thoracic aorta supplying blood to the RUL, one arterial channel seen arising from the arch of aorta supplying blood to the RML and RLL, and another large tortuous arterial channel arising from the descending thoracic aorta supplying the LUL and LLL) (Figures 4 and 6). Also seen was a large anterior 2.4-centimetre sized subaortic VSD, overriding of aorta, and right ventricular hypertrophy confirming the echocardiography findings (Figure 5). The absence of the native pulmonary vascular channels with MAPCAs supplying the lung directly indicates a type C PA-VSD.

Additionally, there was presence of right sided aortic arch with dilated ascending aorta, arch of aorta and descending thoracic aorta. A calcified lesion was noted in the superior segment of the right lower lobe of lung with a pulmonary arterial branch seen supplying it and a draining pulmonary vein adjacent to it suggesting a calcified AVM (Figure 4(b)).

## 3. Discussion

PA-VSD is also known as “pseudotruncus arteriosus” or “truncus arteriosus type 4” [2, 3]. It is classified into 3 types depending upon the source of the pulmonary circulation [2]. In type A, there is presence of the native pulmonary arteries with a PDA supplying blood to them. In type B, there is presence of both the major aortopulmonary collateral arteries (MAPCAs) and the native pulmonary arteries. In type C, only MAPCAs are seen providing the pulmonary blood supply with absent native pulmonary arteries. MAPCAs are arteries that develop to supply blood to the lungs when native pulmonary circulation is underdeveloped. MAPCAs usually arise from the descending aorta but also occasionally can arise from the aortic arch and other systemic arteries like subclavian, the carotid, or even the coronary arteries [3].

In our case, the pulmonary arterial supply was from multiple MAPCAs which further divide to supply all the pulmonary segments on both sides. There was also presence of a right sided aortic arch which is seen in 25% cases of PA-VSD.

Our patient was very unique as she did not develop cyanosis or breathlessness up to 21 years due to the MAPCAs supplying blood to the lungs. The overall life expectancy without surgery is reported to be <50% at 1 year of age and ~8% at 10 years. Our case highlights the fact that occasionally the pulmonary blood flow is just adequate to maintain a nearly normal arterial oxygenation (hence no cyanosis) yet without increasing the pulmonary vascular resistance (secondary to torrential flow due to MAPCAS). However, she has a significant chance of developing pulmonary obstructive vascular disease in later life. So a multistep surgical approach was planned for this patient which comprises unifocalization, that is, unification of the MAPCAs followed by right ventricle to pulmonary artery continuity and closure of the VSD [4].

## Abbreviations

PA-VSD: Pulmonary atresia with ventricular septal defect
TOF: Tetralogy of Fallot
MAPCAs: Major aortopulmonary collateral arteries
AVM: Arteriovenous malformation
VSD: Ventricular septal defect
RUL: Right upper lobe
RML: Right middle lobe
RLL: Right lower lobe
LUL: Left upper lobe
LLL: Left lower lobe.
